# Effects of Protease in Soybean Meal-Reduced Diets on Growth Performance, Nutrient Digestibility, and Intestinal Health of Weaned Piglets

**DOI:** 10.3390/ani14010101

**Published:** 2023-12-27

**Authors:** Junhong Zhang, Chunxiang Zhou, Honglei Zou, Bin Li, Bing Yu, Jun He, Ping Zheng, Xiangbing Mao, Hui Yan, Junqiu Luo, Yuheng Luo, Jinyong Chen, Jie Yu

**Affiliations:** 1 Key Laboratory for Animal Disease-Resistance Nutrition of Sichuan Province, Animal Nutrition Institute, Sichuan Agricultural University, Chengdu 611130, China; 15352489173@163.com (J.Z.); zed_zou991126@163.com (H.Z.); ybingtian@163.com (B.Y.); hejun8067@163.com (J.H.); zpind05@163.com (P.Z.); acatmxb2003@163.com (X.M.); yan.hui@sicau.edu.cn (H.Y.); 13910@sicau.edu.cn (J.L.); luoluo212@126.com (Y.L.); 2 Medical School, Huanghe Science and Technology University, Zhengzhou 450009, China; zcx8008@hhstu.edu.cn; 3 Sichuan Tequ Agriculture and Animal Husbandry Technology Group Co., Ltd., Chengdu 610207, China; binli2020@foxmail.com

**Keywords:** protease, weaning piglets, digestibility, intestinal health

## Abstract

**Simple Summary:**

Protease plays a crucial role in enhancing amino acid utilization in animals. This study was conducted to investigate the effects of exogenous protease in weaned pig diets with low protein. The findings demonstrated that protease added in the diets with a 1.5% reduction in soybean meal contributed to improving intestinal morphology and enhancing nutrient absorption, resulting in better growth performance.

**Abstract:**

This experiment was conducted in weaned piglets to determine the effects of exogenous protease to low soybean meal (SBM) diets on growth performance, diarrhea rate, nutrient digestibility, and intestinal morphology. Seventy-two Duroc × Landrace × Yorkshire weaned barrows (21-day-old, 5.88 ± 0.95 kg) were randomly divided into four treatments with six replicates in each following a 2 × 2 factorial arrangement of SBM levels (0 to 14 d, 9%, 7.5%; 15 to 42 d, 20%, 18.5%) and protease (0 or 150 mg/kg) for a 42-day trial. Fecal samples were collected on days 11 to 14 and 38 to 42 of the experiment, and serum, intestinal tissue, and chyme samples were taken at the end of the experiments. Adding protease in low SBM diets had a significant increase in ADG (*p* < 0.05) and a decrease in F/G (*p* < 0.05). Protease significantly reduced the diarrhea rate (*p* < 0.05). Low SBM level decreased the apparent total tract digestibility (ATTD) of crude protein (CP) and ash (*p* < 0.05) but increased the ATTD of dry matter (DM), ash, organic matter (OM), and CP after the addition of protease (*p* < 0.05). The apparent ileal digestibility (AID) of aspartic acid (Asp), threonine (Thr), serine (Ser), alanine (Ala), lysine (Lys), and total amino acids (AAs) were significantly increased by protease supplementation (*p* < 0.05). Both the SBM-reduced and protease-added diets lead to lower albumin (ALB), albumin/globulin (A/G), and urea nitrogen (UREA) (*p* < 0.05), but greater globulin (GLOB) with low SBM diets (*p* < 0.05). The SBM-reduced and protease-added diets decreased the duodenum pH, respectively (*p* < 0.05). The protease increased the villus:crypt (V:C) in the duodenum and ileum, and ileal villus length (*p* < 0.05). In conclusion, the dietary supplementation of 150 mg/kg protease improved the intestinal health and performance of the weaned piglets and reversed the negative effect of a 1.5% SBM reduction in nutrient utilization, intestinal pH, and intestinal morphological parameters of weaned piglets.

## 1. Introduction

Soybean meal (SBM) is the preferred protein resource in feed industries because of its relatively well-balanced amino acid profile [1]. However, the SBM contains numerous anti-nutritional factors (ANFs), such as soybean lectin, urease, soybean antigenic proteins, etc., which impede digestion and absorption of nutrients, thereby wasting protein resources and even affecting animal performance, especially in young animals [2]. Meanwhile, it is noticeable that excess protein in vivo is associated with feed cost and environmental pollution, thereby increasing emphasis on improving protein utilization while appropriately reducing protein levels in diets [3,4]. Therefore, reducing antigenic proteins and improving amino acids (AAs) utilization in feed is critical to increasing the SBM value and is a pressing issue for feed companies and farmers. A study by Qin and colleagues highlighted the role of AAs balance theory in low-protein diets, conducting an effective way to decrease the dietary crude protein (CP) level simultaneously with the supplementation of essential amino acids (EAAs) for maintaining animal growth performance and product quality [5].

Exogenous proteases have emerged to increase the digestible AAs by hydrolyzing large protein molecules into smaller peptides and AAs for improving protein digestibility and reducing nitrogen emission [6,7,8], contributing to improving the bioavailability of nutrients in ingredients and eliminating ANFs as one of the best ways. Additional findings have proved that 200 mg/kg protease supplementation in the corn-SBM diet could improve the utilization of protein and thus contribute to lower nitrogen excretion to the environment [9]. Further studies have confirmed that the dietary protease may contribute to the improvement of intestinal development, protein digestibility, nutrient transport efficiency, and health status of piglets when fed low-digestible protein sources, thereby increasing the growth performance of weaned piglets [10]. Therefore, plenty of works have identified that dietary protease could increase the AAs digestibility, including improving feed conversion rate and intestinal integrity of newly weaned pigs fed normally [11] even low protein diets [12]. However, the difference in the effect of exogenous protease on the performance of weaned piglets was impacted by protein reduction level and exogenous protease addition in diets. Our group previously conducted a preliminary study and found that the 150 mg/kg or 300 mg/kg protease added in the diets with a 6% reduction in SBM could only partially compensate for the absorption and utilization of digestible amino acids (AAs) [13]. It remains unclear how much protein could be saved when dietary protease is applied. The appropriate dose of protease counterbalance for the lower SBM level has not yet been identified. The present study proposed to explore the effects of protease supplementation in a weaner diet with a less reduced SBM (1.5%), which was conducted by evaluating growth performance, nutrient digestibility, and intestinal morphology in piglets fed soybean meal-reduced diets with or without protease.

## 2. Materials and Methods

All the experimental procedures used in this study were approved by the Institutional Animal Care and Use Committee of Sichuan Agricultural University.

### 2.1. Experimental Animals, Design, Diets and Housing

A total of 72 healthy [Duroc × (Landrace × Yorkshire), 21-day-old] weaned barrows with an average body weight of 5.88 ± 0.95 kg were randomly divided into 4 treatments with 6 replicate pens of 3 pigs per pen following a 2 × 2 factorial arrangement of SBM levels (0 to 14 d, 9%, 7.5%; 15 to 42 d, 20%, 18.5%) and protease (0 or 150 mg/kg) according to a randomized complete block design based on body weight. The basal diets met or exceeded the National Research Council (NRC 2012) nutritional requirements for pigs weighing 7 to 11 kg and 11 to 25 kg (Table 1). The protease RONOZYME^®^ ProAct (75,000 PROT/g) in this study was provided by DSM (China) Limited (Shanghai, China), which is a neutral and alkaline protease from the strain Nocardiopsis showing an impressive degree of protein hydrolysis in a corn–soy diet.

The experiment was conducted at the Swine Research Facility of Animal Nutrition Institute, Sichuan Agricultural University. All the pigs were housed in pens (2.5 × 1.8 m^2^) with slatted plastic floors. The pigs were given ad libitum access to feed and water during the 42-day experiment. Room temperature and air humidity were maintained at 25 °C and 50%, respectively. The weighting was performed on days 1, 14, 28, and 42 after 12 h fasting, and feed intake was taken daily for each pen to calculate separately average daily gain (ADG), average daily feed intake (ADFI), and feed to gain ratio (G:F). The morphology of feces was observed and diarrhea cases were recorded daily per pen throughout the entire experiment. Pigs with feces consistency rated 2 or 3, as described by Liu et al. [14], were considered to have diarrhea and calculated as follows:

Diarrhea rate (%) = number of diarrhea per pen/total number of diarrheas × 100.

### 2.2. Sample Collection

The experimental diets were sampled and then fresh feces on a pen basis (3 piglets) were sampled immediately after pig defecation on days 11 to 14 and 38 to 42 of the experiment. Also, 10 mL of 10% H_2_SO_4_ solution was added evenly per 100 g of feces to fix nitrogen.

One pig with the average body weight (~18.5 kg) from each pen was selected for sampling at the end of the experiment. The blood samples were collected from the anterior vena cava using a vacuum tube without anticoagulant, followed by centrifugation (3500× *g*, 4 °C, 15 min) to obtain serum samples, which were stored at −20 °C until analyzed [15].

Then the same piglets were euthanized after anaesthesia for collecting digesta samples 2 h after the last meal at the end of the experiment. Approximately 2 cm segments of intestinal tissue from the proximal ends of the duodenum, jejunum, and ileum were gently taken to avoid squeezing during operation. Intestinal tissues were preserved separately by treatment in 4% paraformaldehyde solution prior to observation of intestinal morphology including the determination of villus height and crypt depth. The chyme samples from each intestinal segment were collected into a sterile tube by gently massaging the intestinal tract and were immediately frozen in liquid nitrogen and then stored at −80 °C for the following determination of pH, amino acid digestibility, and digestive enzyme activities.

### 2.3. Chemical Analyses

The samples (diets and feces) were dried to constant weight in a forced-air drying oven at 65 °C, and then finely ground and sieved through a 1 mm screen to determine the apparent total tract digestibility (ATTD) of dry matter (DM), gross energy (GE), crude protein (CP), and organic matter (OM) using acid insoluble ash (AIA) as a natural tracer. The standard of AOAC (2006) procedures was used for the determination of CP (990.03), DM (934.01), and ASH (942.15). An automatic adiabatic oxygen bomb calorimeter (Parr Instrument Co., Moline, IL, USA) was applied to measure the gross energy. The AIA was determined using the methods described in our previous report [13]. OM content was calculated as follows:

OM = 1 − ASH.

In addition, the amino acids (AAs) content of feed and ileal chyme was measured by AA-analyzer (L-8900, HITACHI. Tokyo, Japan). ATTD or the apparent ileal digestibility (AID) of a nutrient was then calculated according to the formula below:

Digestibility (%) = (1 − A_1_/A × B/B_1_) × 100

where A = nutrient concentration in diet, A_1_ = nutrient concentration in feces or chyme, B = AIA concentration in diet, and B_1_ = AIA concentration in feces or chyme.

### 2.4. Determination of Blood Metabolites

The concentrations of total protein (TP), urea nitrogen (SUN), albumin (ALB), globulin (GLO), aspartate aminotransferase (AST), and alanine aminotransferase (ALT) in serum were measured using 7020 automatic biochemical analyzers (HITACHI, Japan) following the instruction of previous reference [15].

### 2.5. Determination of Digestive Enzyme Activities

The jejunal chyme was mixed with cold saline solution in a conical flask at the ratio of weight (g): volume (mL) = 1:9, and then centrifuged at 2500× *g* for 10 min (4 °C) to obtain the supernatant for determining the activity of chymotrypsin and trypsin using the assay kit purchased from Nanjing Jiancheng Bioengineering Institute and the assay steps were performed strictly according to the instructions.

### 2.6. Determination of pH of Intestinal Digesta

During intestinal chime collection, a Testo 205 pH/temperature meter (Testo Co., Ltd., West Chester, PA, USA) was used to determine the pH of the duodenal, jejunal, ileal, cecum, and colonic digesta.

### 2.7. Intestinal Morphology

Samples of the duodenum, jejunum, and ileum segments were embedded in paraffin and sectioned, then stained with hematoxylin-eosin for identification of villus height and crypt depth using a micrometer under a microscope [16]. The target areas were selected at 40× magnification under an Eclipse Ci-L camera microscope and analyzed with an Image-Pro Plus 6.0 analysis software for measuring 5 intact villi height and their associated crypt depths in each section separately with mm as the standard unit and the average value was calculated.

### 2.8. Statistical Analysis

The experimental data were organized and analyzed using Excel for statistical purposes. The experimental data were subjected to a multivariate analysis using the General Linear Model in IBM SPSS Statistics 27, employing an interaction effects model to examine various statistical indicators, such as main effects, interaction effects, and estimated marginal means, followed by Tukey’s HSD post hoc test. The results were presented as mean values and standard error of the mean (SEM), and a significance level of *p* < 0.05 was considered statistically significant.

## 3. Results

### 3.1. Growth Performance

As shown in Table 2, low-SBM diets with protease significantly increased ADG during d 15–42 (*p* < 0.05) and decreased F/G in the whole trial (*p* < 0.05) compared to control. The positive effects of protease on the ADG and F/G were significantly observed in d 15–42 at both SBM levels when compared to the diet without protease (*p* < 0.05). There was a significant interaction among experiment phase, SBM level, and protease on ADG, ADFI, and F/G (*p* < 0.05), same as SBM level and protease (*p* < 0.05), as well as for experiment phase and SBM level (*p* < 0.05).

### 3.2. Diarrhea Rate

As shown in Table 3, protease supplementation provided significant diarrhea relief in weaned pigs (*p* < 0.05). As the experiment progressed, a significant drop in diarrhea rate was observed (*p* < 0.05). A significant interaction was observed among experiment phase, SBM level, and protease in relation to diarrhea rate (*p* < 0.05), and the same significant interaction between SBM level and protease (*p* < 0.05) and between phase and SBM level (*p* < 0.05).

### 3.3. Nutrient Digestibilities

As shown in Table 4, low SBM levels had a significant reduction in the ATTD of CP and Ash (*p* < 0.05), while a significant increase in the ATTD of DM, Ash, OM, and CP with protease added (*p* < 0.05). In experiment diets, a significant interaction effect was present in the ATTD of DM, OM, CP, and Ash between SBM level and protease (*p* < 0.05).

As shown in Table 5, no significant interaction was found between SBM level and protease in the AID of all AAs (*p* > 0.05). The AID of Asp, Thr, Ser, Glu, Ala, Val, Met, Ile, Leu, Tyr, and Lys was significantly reduced at low SBM levels (*p* < 0.05), whereas the AID of Asp, Thr, Ser, Ala, Lys, and Total AAs was significantly increased in protease supplementation (*p* < 0.05).

### 3.4. Blood Metabolites

As shown in Table 6, both SBM-reduced and protease-added diets lead to lower ALB, A/G, and UREA (*p* < 0.05), but greater GLOB with low SBM diets (*p* < 0.05). A significant interaction between SBM level and protease was observed in ALB, GLOB, A/G, AST, and UREA (*p* < 0.05).

### 3.5. Digestive Enzyme Activities

As shown in Table 7, there was no significant effect on SBM level and protease in trypsin and chymotrypsin activities (*p* > 0.05), and no significant interaction between SBM level and protease in trypsin and chymotrypsin activities (*p* > 0.05).

### 3.6. Intestinal Digesta pH

As shown in Table 8, SBM-reduced and protease-added diets decreased the duodenum pH (*p* < 0.05), respectively, and the pH of jejunal and cecum was decreased by low SBM diets (*p* < 0.05). A significant interaction between SBM level and protease was shown in the pH of duodenum, jejunum, and cecum (*p* < 0.05).

### 3.7. Intestinal Morphology

As shown in Table 9, the pigs exhibited a significant increase of the crypt depth in the duodenum and jejunum fed low SBM diet (*p* < 0.05), and a decrease of villus length and villus:crypt (V:C) in the duodenum and ileum, respectively (*p* < 0.05). The protease increased the V:C in the duodenum and ileum, the same as the ileal villus length (*p* < 0.05). A significant interaction between SBM level and protease was seen in villus length and V:C in duodenum and ileum (*p* < 0.05).

## 4. Discussion

A deficiency in dietary protein leads to growth retardation and malnutrition in pigs, resulting in weight loss, muscle atrophy, and impaired digestive function, as well as reduced feed conversion and increased feed costs [17]. The previous study demonstrated that a low CP diet with added protease could increase growth performance and CP digestibility of weaned piglets to finishing pigs [18]. As regards in vivo trials, feeding additional protease in post-weaning piglets resulted in few enhancements in growth parameters [19]. Our study showed that weaned pigs fed low SBM diets with protease exhibited a significant improvement in growth performance and was consistent with the previous report [13] which suggested that exogenous proteases may facilitate protein breakdown and improve the digestive process, particularly in animals with impaired digestion or malabsorption. Therefore, incorporating exogenous enzyme preparations in diets with SBM-reduced results in a noteworthy enhancement in the growth performance of weaned piglets.

A review from Stein et al. summarized that enzyme treatment results in the removal of oligosaccharides and most of the antigens from SBM [20] confirming that pig diets with exogenous proteases can effectively enhance protein digestion and absorption, thereby alleviating the strain on the gastrointestinal tract and mitigating symptoms of diarrhea [21]. Proteases can efficiently catalyze the hydrolysis of proteins into smaller peptides and AAs facilitating their absorption and utilization in vivo, thereby relieving intestinal stress caused by nutrient accumulation [6]. Additionally, exogenous proteases also attenuate the protein content within the intestine, consequently inhibiting the proliferation of detrimental microflora to a certain extent and reducing the severity of diarrhea [10], a result consistent with Zhu et al. [19]. The present study demonstrated the lower diarrhea occurrence in protease-supplemented pigs supporting the hypothesis that the exogenous proteases may offer potential relief for diarrhea symptoms in specific circumstances.

In a study by Lindemann et al. [22], weanling piglets experienced a severe and rapid drop in endogenous protease (trypsin and chymotrypsin) secretion after weaning, thereby providing a possibility of an increase in the flow of undigested protein to the hindgut, while protease inclusion in piglet feeds could reduce nutrient losses and enhanced the AAs utilization [18,19,21] which is in line with the present study revealing that protease significantly contributed to the utilization of DM, CP, Ash, OM, and even the most AAs. This result proved that the more additional digestible AAs released met the EAA requirements of weaned pigs consuming low-protein diets. Alternatively, adding exogenous enzymes (such as protease) to post-weaning diets can aid in compensating for underdeveloped endogenous enzyme secretory capacity and enhancing nutrient digestibility in weaned pigs [23]. Further researchers confirmed that adding protease could improve nutrient utilization thus positively impacting growth performance in weaned pigs, even in low-protein diets [12]. However, in the present work, the AA digestibility improvement was observed in weaned piglets fed low SBM diets supplemented with exogenous proteases which was still significantly lower than that of pigs fed normal SBM diets. Thus, the main issue is related to the digestible amino acids released may not fully compensate for the deficiency in some EAAs probably due to a reduction of dietary SBM level [9].

The study found a positive correlation between CP concentration and serum urea concentration [24], and it was observed a decrease in dietary protein levels has a corresponding decrease in serum urea levels in piglets [25,26], which is consistent with the results of this study. Blood serum urea nitrogen concentration can be used as an indicator of protein status including nitrogen utilization problems in animal treatments [27]. However, the incorporation of protease increased the amount of available small peptides and AAs and decreased the ANFs so that the serum UREA levels in piglets were lower than those of piglets supplemented without protease. These results are consistent with the effect of protease on the ATTD of crude protein suggesting that protease may play a role in enhancing dietary protein utilization by weaned piglets. Furthermore, the reduction in protein levels was accompanied by significant changes in ALB and A/G in this study, whereas ALB is mainly produced by the liver [28], which could be a sign of a reduced raw material for the synthesis of ALB or concern for impaired hepatic and renal function of the piglets.

Digestive enzymes in vivo need to be activated for optimal function [29], and exogenous proteases play a role in facilitating the activation of these enzymes. Our previous work found that the inclusion of 300 mg/kg protease in a diet resulted in an elevation in the activity of trypsin and chymotrypsin in the jejunum, but no significant improvement with 150 mg/kg protease [19]. Thus, based on a dietary protein level comparable to that of the previous one [19], including 150 mg/kg protease in the present research agreed with the previous findings [19]. Secondly, consistent with the results of the present study, reducing dietary SBM may reduce undigested protein in the piglet hindgut, thereby decreasing the production of ammonia and contributing to a reduction in intestinal pH [22]. A previous study showed that a decrease in intestinal pH could eliminate the favorable growing conditions for neutral and aerobic bacteria, consequently hindering the growth of pathogenic bacteria [30]. Additionally, by promoting protein digestion and absorption in the small intestine, proteases could reduce the production of ammonia in the hindgut, effectively lowering the pH of the intestinal tract in this study, which is consistent with the previous research [19,31], hypothesizing and demonstrating that proteases possess the ability to enhance the proliferation of beneficial bacteria in the intestinal tract of piglets, thereby facilitating improving their production performance due to maintain intestinal normal physiological functions.

Increased amounts of undigested protein in the hindgut of weanling piglets adversely affected intestinal health and digestive capacity [22], while proteases are described as one of the most economically relevant feed additives in maintaining intestinal physiological health [32,33]. Proteases exert important effects on intestinal morphology through diverse pathways in a recent study. Specifically, proteases facilitate the proliferation and differentiation of intestinal epithelial cells to augment the abundance of intestinal epithelial cells and the thickness of the intestinal wall, as well as repair and regeneration of the intestinal mucosa for mitigating inflammation and minimizing damage to the intestinal mucosa [34]. Following weaning, the switch from liquid to solid diets damages the intestinal villi, impairing intestinal absorption and digestive enzyme secretion [35,36], which can seriously affect nutrient absorption and the overall health status of piglets. The proteases have been employed to promote the growth and repair of enterocytes and increase the height of intestinal villi and the thickness of the intestinal mucosa, potentially improving nutrient absorption and preventing the invasion of pathogenic bacteria [37]. This aligns with the findings of the present study.

In conjunction with the aforementioned findings, an appropriate amount of protease added in SBM-reduced diets for weaned piglets has been positively correlated with growth promotion, diarrhea reduction, and intestinal health. The intestinal tract is an important site for protein digestion and absorption where the protease may protect intestinal morphology from stress, in addition to maintaining a more favorable intestinal environment for protein catabolism and efficient utilization. However, the AA balance should be taken into account when reducing the SBM levels to avoid nutritional problems in piglets.

## 5. Conclusions

In conclusion, the low SBM level appeared to be effective in alleviating diarrhea, but a 1.5% reduction in SBM negatively affected nutrient utilization, intestinal pH, and intestinal morphological parameters in weaned piglets. Adding 150 mg/kg protease to low SBM diets could further promote weaned piglet intestinal health and performance.

## Figures and Tables

**Table 1:** Ingredients and nutrient composition of the experimental diets (as-fed basis).

Item	Phase I (d 1–14)		Phase II (d 15–42)	
	Normal	Low	Normal	Low
Ingredient, %				
Corn (CP 7.8%)	31.20	32.65	66.00	67.40
Corn, extruded (CP 7.8%)	27.00	27.00		
Soybean meal (46%)	9.00	7.50	20.00	18.50
Soybean meal extruded	8.00	8.00		
Soy protein concentrate	7.00	7.00	1.50	1.50
Fish meal (CP 62.5%)	3.00	3.00	3.00	3.00
Whey powder (CP 3%)	8.00	8.00	3.00	3.00
Sucrose	2.00	2.00	2.00	2.00
Soybean oil	1.70	1.60	1.70	1.60
Choline chloride	0.10	0.10	0.10	0.10
NaCl	0.30	0.30	0.30	0.30
Vitamin premix ^a^	0.05	0.05	0.05	0.05
Mineral premix ^b^	0.30	0.30	0.30	0.30
Limestone	0.75	0.75	0.70	0.71
Dicalcium phosphate	0.85	0.86	0.72	0.75
L-Lysine-HCl	0.44	0.44	0.37	0.37
DL-Methionine	0.16	0.16	0.14	0.14
L-Threonine	0.14	0.11	0.11	0.08
L-Tryptophan	0.01	0.01	0.01	0.01
Nutrient levels ^c^				
DE, MJ/kg	14.78	14.78	14.32	14.32
CP, %	18.82	18.24	17.81	17.23
Ca, %	0.80	0.80	0.70	0.70
Total P, %	0.65	0.65	0.60	0.60
Available P, %	0.46	0.46	0.39	0.40
DLys, %	1.35	1.31	1.23	1.19
DMet, %	0.46	0.45	0.43	0.42
DMet + DCys, %Dcys(%)	0.74	0.72	0.68	0.66
DThr, %	0.79	0.76	0.73	0.70
DTrp, %	0.22	0.21	0.20	0.20

^a^ Vitamin premix provided the following quantities of vitamins per kilogram of a complete diet: vitamin A, 15,000 IU; vitamin D_3_, 5000 IU; vitamin E, 40 IU; vitamin K_3_, 5 mg; vitamin B_1_, 5 mg; vitamin B_2_, 12.5 mg; vitamin B_6_, 6 mg; vitamin B_12_, 0.06 mg; niacin, 50 mg; folic acid, 2.5 mg; biotin, 0.25 mg; pantothenic acid, 25 mg. ^b^ Mineral premix provided the following quantities of minerals per kilogram of complete diet: Fe 100 mg (FeSO_4_·H_2_O); Cu 6 mg (CuSO_4_·5H_2_O); Mn 4 mg (MnSO_4_·H_2_O); Zn 100 mg (ZnSO_4_·H_2_O), 1600 mg (ZnO); I 0.14 mg (KI); Se 0.30 mg (Na_2_SeO_3_). ^c^ Nutrient levels are theoretical values except the CP was measured value.

**Table 2:** Effect of protease on performance in weaned pigs with different SBM levels.

SBM	Protease	ADG (g)					ADFI (g)					F/G				
		d 1–14	d 15–28	d 29–42	d 15–42	d 1–42	d 1–14	d 15–28	d 29–42	d 15–42	d 1–42	d 1–14	d 15–28	d 29–42	d 15–42	d 1–42
Normal	0 mg/kg	95.23 ^ab^	285.60 ^a^	527.46 ^a^	406.53 ^ab^	296.58 ^a^	227.13 ^a^	551.12 ^ab^	958.80 ^ab^	754.96 ^ab^	575.02 ^ab^	2.77 ^a^	2.04 ^c^	1.84 ^c^	1.87 ^c^	1.96 ^c^
	150 mg/kg	101.45 ^b^	339.86 ^b^	534.11 ^a^	436.98 ^b^	323.51 ^b^	248.24 ^b^	610.89 ^c^	950.33 ^ab^	780.61 ^b^	603.15 ^b^	3.29 ^b^	1.80 ^b^	1.78 ^b^	1.79 ^b^	1.88 ^b^
Low	0 mg/kg	89.90 ^ab^	290.05 ^a^	517.37 ^a^	401.97 ^a^	294.27 ^a^	226.71 ^a^	532.07 ^a^	926.71 ^a^	729.39 ^a^	561.83 ^a^	2.88 ^a^	1.85 ^b^	1.78 ^b^	1.81 ^b^	1.91 ^bc^
	150 mg/kg	80.18 ^a^	351.36 ^b^	584.47 ^b^	467.91 ^c^	341.49 ^b^	215.15 ^a^	573.80 ^ab^	1004.33 ^b^	789.07 ^b^	601.75 ^b^	2.78 ^a^	1.64 ^a^	1.71 ^a^	1.68 ^a^	1.77 ^a^
SEM		5.18	7.83	10.47	8.35	7.12	5.21	12.29	19.89	15.73	11.94	0.14	0.03	0.02	0.01	0.02
*p*-Values																
SBM		0.003					0.192					0.011				
Protease		0.004					0.225					<0.001				
phase		<0.001					<0.001					<0.001				
SBM×Protease		<0.001					<0.001					0.035				
Phase×SBM		0.012					0.023					0.001				
Phase×Protease		0.562					0.406					0.351				
Phase×SBM×Protease		0.002					<0.001					<0.001				

Normal = normal soybean meal diet, Low = low soybean meal diet; SEM = standard error of means; SBM = soybean meal. Within a column, the mean values with different letters differ at *p* < 0.05.

**Table 3:** Effect of protease on diarrhea rate in weaned piglets fed diets with different SBM levels.

Item	Normal		Low		SEM	*p*-Value						
	0 mg/kg	150 mg/kg	0 mg/kg	150 mg/kg		SBM	Protease	Phase	SBM×Protease	Phase×SBM	Phase×Protease	Phase×SBM×Protease
d 1–14	51.52 ^b^	51.39 ^b^	46.44 ^b^	33.68 ^a^	2.04	0.164	<0.001	<0.001	<0.001	<0.001	0.626	<0.001
d 15–28	30.93 ^b^	18.68 ^a^	18.83 ^a^	13.70 ^a^	1.90							
d 29–42	14.31 ^b^	6.08 ^a^	8.46 ^a^	7.09 ^a^	1.44							
d 15–42	22.62 ^b^	12.38 ^a^	13.64 ^a^	10.39 ^a^	1.61							
d 1–42	30.77 ^c^	22.38 ^b^	22.89 ^b^	16.96 ^a^	1.63							

Normal = normal soybean meal diet, Low = low soybean meal diet; SEM = standard error of means; SBM = soybean meal. Within a row, the mean values with different letters differ at *p* < 0.05.

**Table 4:** Effect of protease on ATTD of nutrients in weaned pigs fed diets with different SBM levels.

Item, %	Normal		Low		SEM	*p*-Value		
	0 mg/kg	150 mg/kg	0 mg/kg	150 mg/kg		Protease	SBM	SBM×Protease
DM	76.34 ^bc^	82.89 ^c^	67.17 ^a^	73.35 ^ab^	2.70	<0.001	0.946	0.020
ASH	52.70 ^b^	55.33 ^c^	51.38 ^a^	52.09 ^ab^	0.31	0.002	<0.001	<0.001
OM	76.55 ^bc^	82.81 ^c^	66.73 ^a^	73.59 ^ab^	2.85	0.001	0.917	0.024
CP	58.17 ^a^	60.57 ^b^	57.73 ^a^	57.89 ^a^	0.54	0.004	0.019	0.039
GE	79.59	79.74	77.92	79.51	1.33	0.518	0.591	0.483

Normal = normal soybean meal diet, Low = low soybean meal diet; SEM = standard error of means; SBM = soybean meal; DM = dry matter; OM = organic matter; CP = crude protein; GE = gross energy. Within a row, the mean values with different letters differ at *p* < 0.05.

**Table 5:** Effect of protease on SID of amino acids in weaned pigs fed diets with different SBM levels.

Item, %	Normal		Low		SEM	*p*-Value		
	0 mg/kg	150 mg/kg	0 mg/kg	150 mg/kg		Protease	SBM	SBM×Protease
Asp	56.50 ^b^	59.75 ^c^	53.01 ^a^	57.72 ^bc^	1.06	0.011	<0.001	0.492
Thr	61.89 ^b^	68.62 ^c^	54.33 ^a^	56.89 ^a^	1.39	0.001	<0.001	0.135
Ser	67.70 ^c^	74.15 ^d^	54.91 ^a^	60.72 ^b^	1.81	<0.001	<0.001	0.861
Glu	67.59 ^b^	72.70 ^b^	56.27 ^a^	57.26 ^a^	2.00	0.130	<0.001	0.305
Gly	57.27	60.48	55.58	55.78	2.91	0.606	0.275	0.559
Ala	53.78 ^a^	59.09 ^b^	52.58 ^a^	53.27 ^a^	1.46	0.018	0.042	0.115
Cys	68.65	68.77	65.36	67.63	3.24	0.713	0.496	0.740
Val	74.26 ^bc^	76.73 ^c^	66.19 ^a^	69.59 ^ab^	2.06	0.158	<0.001	0.823
Met	63.72 ^b^	68.13 ^b^	52.46 ^a^	54.45 ^a^	2.46	0.196	<0.001	0.623
Ile	68.95 ^b^	71.38 ^b^	63.17 ^a^	70.57 ^b^	2.04	0.109	0.018	0.226
Leu	63.73 ^a^	75.59 ^b^	62.91 ^a^	70.20 ^b^	2.19	0.158	<0.001	0.299
Tyr	71.67 ^b^	75.65 ^b^	65.51 ^a^	73.58 ^b^	2.12	0.054	0.005	0.337
Phe	85.77	86.93	83.29	84.77	2.50	0.599	0.354	0.950
Lys	73.43 ^b^	77.67 ^c^	66.42 ^a^	70.65 ^ab^	1.74	0.016	<0.001	0.997
His	83.53	84.71	81.42	83.12	2.05	0.483	0.368	0.899
Arg	65.98	68.53	64.85	65.55	1.51	0.284	0.176	0.539
Pro	53.30	55.70	51.51	55.17	1.64	0.481	0.067	0.702
Total	64.67 ^ab^	69.78 ^b^	62.75 ^a^	69.08 ^b^	1.79	0.002	0.465	0.733

Normal = normal soybean meal diet, Low = low soybean meal diet; SEM = standard error of means; SBM = soybean meal. Within a row, the mean values with different letters differ at *p* < 0.05.

**Table 6:** Effect of protease on blood metabolites in weaned pigs fed diets with different SBM levels.

Item	Norma		Low		SEM	*p*-Value		
	0 mg/kg	150 mg/kg	0 mg/kg	150 mg/kg		Protease	SBM	SBM×Protease
TP (g/L)	46.50 ^ab^	46.96 ^b^	45.43 ^a^	46.01 ^ab^	0.41	0.213	0.016	0.889
ALB (g/L)	30.50 ^b^	27.55 ^a^	27.52 ^a^	26.48 ^a^	0.44	0.032	<0.001	<0.001
GLOB (g/L)	16.46 ^a^	17.88 ^ab^	18.98 ^bc^	19.61 ^c^	0.43	0.361	0.02	<0.001
A/G	2.10 ^b^	1.55 ^a^	1.45 ^a^	1.36 ^a^	0.11	0.038	0.004	<0.001
ALT (U/L)	49.32 ^ab^	59.11 ^c^	43.98 ^a^	50.71 ^b^	1.55	<0.001	<0.001	0.325
AST (U/L)	64.15 ^b^	37.55 ^a^	32.12 ^a^	59.41 ^b^	3.19	0.113	0.914	<0.001
BUN (mmol/L)	2.85 ^c^	2.41 ^b^	2.00 ^a^	1.87 ^a^	0.04	<0.001	<0.001	<0.001

Normal = normal soybean meal diet, Low = low soybean meal diet; SEM = standard error of means; SBM = soybean meal; TP = total protein, ALB = albumin, GLOB = globulin, A/G = albumin/globulin, ALT = Alanine Aminotransferase, AST = Aspartate Aminotransferase, BUN = Blood Urea Nitrogen. Within a row, the mean values with different letters differ at *p* < 0.05.

**Table 7:** Effect of protease on jejunum digestive enzyme activities in weaned piglets fed a diet with different SBM levels.

Item, U/mg Prot	Normal		Low		SEM	*p*-Value		
	0 mg/kg	150 mg/kg	0 mg/kg	150 mg/kg		Protease	SBM	SBM×Protease
Chymotrypsin	34.79	42.02	26.81	34.85	7.53	0.314	0.318	0.957
Trypsin	7433.36	8043.80	6807.55	7477.66	1576.93	0.687	0.707	0.985

Normal = normal soybean meal diet, Low = low soybean meal diet; SEM = standard error of means; SBM = soybean meal.

**Table 8:** Effect of protease on intestinal pH in weaned piglets fed diet with different SBM levels.

Item	Normal		Low		SEM	*p*-Value		
	0 mg/kg	150 mg/kg	0 mg/kg	150 mg/kg		Protease	SBM	SBM×Protease
Duodenum	6.68 ^d^	5.79 ^c^	5.57 ^b^	5.35 ^a^	0.01	<0.001	<0.001	<0.001
Jejunum	6.27 ^b^	6.10 ^a^	6.01 ^a^	6.01 ^a^	0.04	0.055	<0.001	0.046
Ileum	6.37 ^b^	6.22 ^a^	6.30 ^ab^	6.31 ^ab^	0.04	0.127	0.829	0.088
Cecum	5.58 ^b^	5.71 ^c^	5.53 ^ab^	5.47 ^a^	0.03	0.218	<0.001	0.003
Colon	5.96	6.00	5.93	5.96	0.04	0.353	0.283	0.817

Normal = normal soybean meal diet, Low = low soybean meal diet; SEM = standard error of means; SBM = soybean meal; within a row, mean values with different letters differ at *p* < 0.05.

**Table 9:** Effect of protease on intestinal morphology in weaned piglets fed a diet with different SBM levels.

Item	Normal		Low		SEM	*p*-Value		
	0 mg/kg	150 mg/kg	0 mg/kg	150 mg/kg		Protease	SBM	SBM×Protease
Duodenum								
Villi length (µm)	455.30 ^b^	509.93 ^c^	377.68 ^a^	449.03 ^b^	16.09	0.604	<0.001	<0.001
Crypt depth (µm)	224.04 ^b^	188.54 ^a^	233.77 ^b^	234.33 ^b^	12.18	0.141	0.024	0.154
V:C Ratio	2.14 ^a^	3.06 ^b^	1.87 ^a^	2.08 ^a^	0.15	0.017	<0.001	<0.001
Ileum								
Villi length (µm)	448.03 ^a^	556.03 ^b^	415.94 ^a^	424.01 ^a^	16.84	<0.001	0.004	<0.001
Crypt depth (µm)	197.18 ^a^	196.20 ^a^	228.91 ^b^	190.27 ^a^	9.60	0.181	0.052	0.041
V:C Ratio	2.57 ^b^	2.99 ^c^	1.97 ^a^	2.37 ^ab^	0.15	0.017	<0.001	<0.001
Jejunum								
Villi length (µm)	442.54 ^b^	449.10 ^b^	438.69 ^a^	446.67 ^b^	14.84	0.962	0.625	0.833
Crypt depth (µm)	172.38 ^ab^	156.93 ^a^	183.38 ^b^	151.31 ^a^	8.43	0.326	0.006	0.750
V:C Ratio	2.76 ^ab^	3.22 ^b^	2.61 ^a^	3.06 ^ab^	0.16	1.000	0.004	0.323

Normal = normal soybean meal diet, Low = low soybean meal diet; SEM = standard error of means; SBM = soybean meal. Within a row, the mean values with different letters differ at *p* < 0.05.

## Data Availability

The data are available on request from the corresponding author.
